# Japan National University Hospitals Infection Control Conference (JNUHICC) report: data summary of device-associated infections

**DOI:** 10.1186/1753-6561-5-S6-P213

**Published:** 2011-06-29

**Authors:** T Watanabe, S Takakura, Y Tanabe, K Yoshio, S Arakawa, S Ichiyama

**Affiliations:** 1 Japan National University Hospitals Infection Control Conference, Nagoya, Japan

## Introduction / objectives

To aggregate separate information into a national database and to enhance quality improvement activities, JNUHICC Sectional Meeting of Surveillance has created a data collection system for device-associated infection rates.

## Methods

Data on CLABSI, VAP, and CAUTI occurring between July 2009 and September 2010 were collected from national university hospitals in Japan and were analyzed. A uniform protocol was constructed in accordance with the definitions of NHSN. CLABSI surveillance during the latter study period (from January to September 2010) included data on clinical sepsis (CSEP) and criterion 2-b (one positive blood culture with common skin contaminant and antimicrobial therapy), and the validity of this addition was assessed.

## Results

CLABSI data were reported from 20 ICUs, 21 hematology/oncology wards, and 14 gastroenterological surgery wards. The mean rates were 1.14, 2.10, and 1.66 per 1000 device-days (DD), respectively. The rates on the data with the inclusion of CSEP and criterion 2-b were1.93, 2.49, and 2.89, respectively. The VAP rate (reported from 27 ICUs) was 2.38 per 1000 DD. The CAUTI rate (reported from 11 ICUs) was 2.40 (SD 8.84) per 1000 DD. The rate and SD decreased 0.92 and 1.09, respectively, when cases only with asymptomatic bacteriuria (ASB), which consisted of 62% of the CAUTI cases, were excluded.

## Conclusion

Indicative and representative values for three major device-associated infection rates at national university hospitals in Japan were obtained. To continue the multi-institutional surveillance of constant quality, analyzing the summary data and reviewing the protocols may be essential.

## Disclosure of interest

None declared.

